# Influence of the homotopy stability perturbation on physical variations of non-local opto-electronic semiconductor materials

**DOI:** 10.1007/s12200-024-00141-3

**Published:** 2024-12-06

**Authors:** A. El-Dali, Mohamed I. A. Othman

**Affiliations:** 1 Department of Mathematics, Faculty of Science, HelwanUniversity, Cairo, 11795 Egypt; 2 Department of Mathematics, Faculty of Science, Zagazig University, Zagazig, 44519 Egypt

**Keywords:** Homotopy perturbation method, Opto-electronic deformation, Laplace transform, Non-local excitation, Bifurcation solutions, Stability perturbation

## Abstract

In the current work, we investigate a novel technique specialized in stability perturbation theory to analyze the primary variations such as thermal, carrier, elastic, and mechanical waves in photothermal theory. The interface of the non-local semiconductor material is utilized to study the stability analysis. The problem is established using a 1D opto-electronic-thermoelastic deformation in the context of the photo-thermoelasticity (PTE) framework. The Laplace transform method is used to convert the system from the time domain into the frequency domain, and the boundary conditions for the thermal, elastic, and plasma waves are applied to the interface of the medium. The homotopy perturbation method was used as an innovative approach to analyze the stability of the non-local silicon’s primary physical fields. The numerical inversion method is applied, yielding many graphs focusing on important numerical factors such as non-local effects, thermo-energy, and thermo-electric coupling parameters. Investigating dual solutions between stable and unstable regions for critical parameters like thermo-electric and thermo-energy coupling factors demonstrates that the homotopy perturbation technique can effectively analyze the stability analysis. The comparison between silicon and germanium is illustrated graphically. Utilizing the homotopy perturbation technique, we can effectively examine the stability of the primary physical variations with the effect of some values for eigenvalues approaches.

**Graphical abstract:**

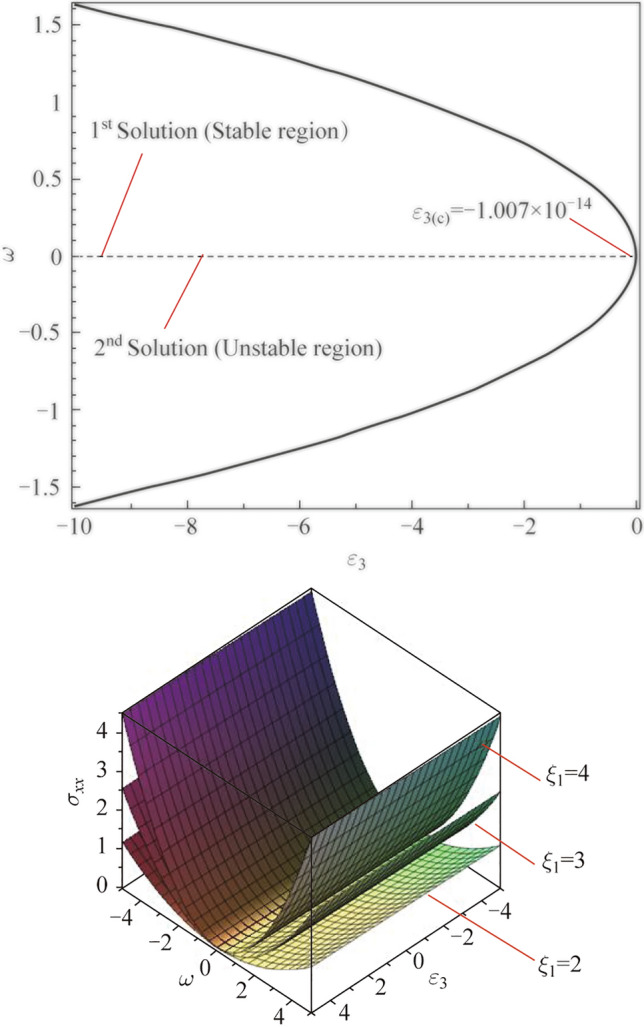

## Introduction

The homotopy perturbation method (HPM) is a particular case of the original homotopy analysis method (HAM). Fundamentally, both methods rely on Taylor series expansions concerning an embedding parameter. Additionally, HPM and HAM can yield highly accurate approximations with just a few terms, provided that the initial guess and the chosen auxiliary linear operator are sufficiently precise. A new perturbation method has been introduced, differing from traditional ones by not requiring a small parameter in the equation. This method uses the homotopy technique by creating a homotopy with an embedding parameter $p \in [0,1]$. The embedding parameter is treated as a “small parameter”; hence, the method is named the HPM. This note concisely introduces the HPM solution procedure, focusing on constructing an appropriate homotopy equation [1, 2]. Liao [3] introduced the powerful HAM as an analytical approach for addressing nonlinear problems. Liao [1] conducts a comparative study of two prominent analytical techniques for solving nonlinear problems: the HAM and the HPM. Watson and Haftka [4] provide a comprehensive overview of modern homotopy methods in optimization, detailing their theoretical foundations, algorithmic development, and practical applications.

Recently, many researchers have been interested in the photo-thermoelasticity (PTE) theory and its applications. This theory describes the interactions between optical, mechanical, and thermal waves in materials subjected to light-induced heating. The photon absorbed by a material causes thermal expansion or contraction and generates mechanical stresses, leading to the material’s deformation. Many authors [5–9] have studied semiconductor materials using the PTE theory. El-Sapa et al. [10] explained how the variation of the thermal conductivity affects the micro elongation and the rotation factors during photo-thermal excitations. Abouelregal et al. [11] study a viscoelastic microbeam that is stressed initially and considers the laser pulse using photo-thermo-elasticity with a two-phase lag effect. Considering the memory effect, Sur [12] investigates the photo-thermoelastic interaction in a semiconductor material containing a cylindrical cavity. Ezzat et al. [13–17] provided a new solution in generalized thermoelasticity theory utilizing a series of functions, contributing to the understanding of PTE theory.

The non-local effects have helped us understand the uniform complex interactions between thermal and mechanical waves. The non-local semiconductor medium in photo-thermal interaction is crucial for predicting stress concentration and wave dispersion in advanced materials. Many authors [18–25] investigated the theory of photo-thermo-elasticity using the non-local semiconductor medium. Kaur and Singh [26] investigated the behavior of a photo-thermoelastic semiconductor resonator using nonlocal memory-dependent derivative analysis. Hosseini et al. [27] represents the study of the non-local semiconductor medium, considering that the nano/microbeam resonator was subjected to plasma shock loading. The non-local theory with the effect of photo-thermal-excitation in a semiconductor material has been studied by Sardar et al. [28]. Ezzat et al. [29–34] have examined many problems in generalized thermoelasticity theory, especially in a non-local semiconductor medium. Their valuable work has led scientists to develop and understand the non-local PTE theory. Saeed and Abbas [35] investigated the effects of a nonlocal thermoelastic model on a thermoelastic nano-scale material. The reflection of thermoelastic waves in semiconductor nanostructures within a non-local porous medium was studied by Ali et al. [36].

The paper aims to focus on the novelty of the stability perturbation analysis on the primary fields of the non-local semiconductor material. Investigating the stable and unstable region (bifurcation solutions) using the eigenvalue approach for the crucial parameters utilizing a homotopy perturbation method is considered a new technique in PTE theory. Seydel [37] focused on the theory and practical application of bifurcation and stability analysis in nonlinear dynamical systems. Hassanin et al. [38] explained the stability perturbation analysis on the physical variations during the photo-thermal excitation. Bhattacharyya [39] explored dual solutions in boundary layer stagnation-point flow and mass transfer with a chemical reaction over a stretching/ shrinking sheet. Many authors [40–43] investigated the presence of dual solutions in mixed convection flow through a porous medium and analyze the bifurcation solutions in a shrinking sheet’s magneto-hydrodynamic (MHD) stagnation-point flow.

## Mathematical formulations

Thermal waves (temperature distribution), optoelectronic waves (carrier distribution), and elastic waves (displacement distribution) are the main physical variations that describe the medium of the non-local semiconductor in our problem. Using the fundamental principles of conversation laws of thermodynamics and the constitutive relations, one can obtain the controlling system of the coupled phenomenon between thermal–mechanical-optoelectronic waves as the following [8–10]:

1 $$\frac{\partial N}{{\partial t}} = D_{E} \nabla^{2} N - \,\frac{N}{\tau } + \kappa \;T,$$

2 $$\rho \,C_{e} \frac{{\partial T(\mathop{r}\limits^{\rightharpoonup} ,t)}}{\partial t} = k\nabla^{2} T(\mathop{r}\limits^{\rightharpoonup} ,t) - \,\frac{{E_{g} }}{\tau }N(\mathop{r}\limits^{\rightharpoonup} ,t) + \gamma T_{0} \nabla .\frac{{\partial \vec{u}(\mathop{r}\limits^{\rightharpoonup} ,t)}}{\partial t}\,,$$

3 $$\rho \,(1 - \xi_{1}^{2} \nabla^{2} )\frac{{\partial^{2} \vec{u}(\mathop{r}\limits^{\rightharpoonup} ,t)}}{{\partial t^{2} }} = \mu \nabla^{2} \vec{u}(\mathop{r}\limits^{\rightharpoonup} ,t) + \,(\mu + \lambda )\,\nabla \,[\nabla .\,\vec{u}(\mathop{r}\limits^{\rightharpoonup} ,t)] - \gamma \nabla T(\mathop{r}\limits^{\rightharpoonup} ,t) - \delta_{n} \nabla N(\mathop{r}\limits^{\rightharpoonup} ,t).$$

Additionally, the non-local stress–strain equation can take the form [23, 24]:

4 $$\left. \begin{gathered} (1 - \xi_{1}^{2} \nabla^{2} )\sigma_{ij} = \sigma_{ij}^{\prime } \,\,\,\, \hfill \\ \sigma_{ij} = \mu (u_{i,j} + u_{j,i} ) + (\lambda \;u_{k,k} - \gamma \,T\, - \gamma_{n} N)\delta_{ij} \hfill \\ \end{gathered} \right\},$$

where $D_{E} ,\,\,\tau ,\,\,E_{g} ,\,\,\mu ,\,\,\lambda ,\,\,\rho ,\,\,k,\,\,{\text{and}}\;T_{0}$ represent the physical constants of thermal-optical-elastic properties, which can be defined as the carrier’s diffusion coefficient, the lifetime relaxation time, the gap energy, the Lame’s constants for elastic medium, the density, the thermal conductivity, and the reference temperature, respectively. $\kappa = \frac{{\partial N_{0} }}{\partial T}\frac{T}{\tau }$ is a parameter describing the nonzero activation coupling case at the equilibrium carrier concentration $N_{0} .$ The volume thermal expansion is $\gamma_{n} = (3\lambda + 2\mu )\alpha_{T} ,$ $\alpha_{T}$ represents the linear thermal expansion parameter. $C_{e}$ is the specific heat coefficient, $\delta_{n} = (3\lambda + 2\mu )d_{n}$ is the difference between the valence band and conductive potential, and $d_{n}$ is electronic deformation coefficient. Considering a one-dimension problem in the direction of *x*-axis, the displacement vector can take the form $(\vec{u}(x,t),0,0),$ and for more simplicity to our non-local coupled system, the non-dimensional property for the 1D problem can take the following form:

5 $$(x^{\prime},\;u^{\prime},\xi_{1}^{\prime } ) = \frac{{(x,\;u,\xi_{1} )}}{{C_{T} t^{*} }},\,\,\,t^{\prime} = \frac{t}{{t^{*} }},\,\,\,(T^{\prime},N^{\prime}) = \frac{{(\gamma T,\gamma_{n} N)}}{2\mu + \lambda },\,\,\,\sigma^{\prime} = \frac{\sigma }{\mu }.$$

Dropping the dashed notation from all physical variations after substituting with Eq. (5), the non-local coupled system (Eqs. (1)–(4)) can take the following form:

6 $$\left( {\frac{{\partial^{2} }}{{\partial x^{2} }} - A_{1} \frac{\partial }{\partial t} - A_{2} } \right)N + \varepsilon_{3} \,T = 0,$$

7 $$\left( {\frac{{\partial^{2} }}{{\partial x^{2} }} - \,\frac{\partial }{\partial t}} \right)T - \alpha_{2} N + \alpha_{3} \left( {\frac{\partial }{\partial t}\frac{\partial u}{{\partial x}}} \right) = 0,$$

8 $$\left[ {\frac{{\partial^{2} }}{{\partial x^{2} }} - \,\left( {1 - \xi_{1}^{2} \frac{{\partial^{2} }}{{\partial x^{2} }}} \right)\frac{{\partial^{2} }}{{\partial t^{2} }}} \right]\,u - \frac{\partial T}{{\partial x}}\, - \,\frac{\partial N}{{\partial x}} = 0,$$

9 $$\sigma { = }\sigma_{xx} = \alpha_{4} \left[ {\frac{\partial u}{{\partial x}} - (\,T\, + N)} \right],$$

where

$$\,A_{1} = \frac{k}{{D_{E} \rho C_{e} }},\,\,\,A_{2} = \frac{{kt^{*} }}{{D_{E} \rho \tau C_{e} }},\,\,\quad \,\varepsilon_{3} = \frac{{d_{n} k\kappa t^{*} }}{{\alpha_{T} \rho C_{e} D_{E} }}\,,\,\,\quad \,\alpha_{2} = \frac{{\alpha_{T} E_{g} t^{*} }}{{d_{n} \rho \tau C_{e} }},\,\,\quad \,\alpha_{3} = \frac{{\gamma^{2} T_{0} \,t^{*} }}{\rho },\,t^{*} = \frac{k}{{\rho C_{e} C_{T}^{2} }},\,\;C_{T}^{2} = \frac{2\mu + \lambda }{\rho }.$$

Here, $\alpha_{3} ,$ $\alpha_{2}$, and $\varepsilon_{3}$ can be named as thermo-elastic coupling parameter, thermo-energy coupling parameter, and thermo-electric coupling parameter, respectively.

## Solving the problem

The Laplace transformation method effectively solves our problem by transferring the partial differential equations (PDEs) to ordinary differential equations (ODEs) for any function $\prod (x,t)$ by applying the following formula [38]:

10 $$L(\prod \,(x,\,t)) = \overline{\prod }(x,\,s) = \int\limits_{0}^{\infty } {\prod \,\,(x,\,t)\,{\text{e}}^{ - \,st} } \,{\text{d}}\,t.$$

With the following initial conditions and the homogeneity of the system

$$\left. {u(x,\,t)} \right|_{t = 0} = \,\,\,\left. {\frac{\partial u(x,\,t)}{{\partial t}}} \right|_{t = 0} = 0\,,\,\,\,\left. {T(x,t)} \right|_{t = 0} = \left. {\frac{\partial T(x,\,t)}{{\partial t}}} \right|_{t = 0} = 0\,,\,\,\,\left. {\sigma (x,t)} \right|_{t = 0} = \left. {\frac{\partial \sigma (x,\,t)}{{\partial t}}} \right|_{t = 0} = 0\,,\,\,$$

11 $$\,\left. {N(x,t)} \right|_{t = 0} = \left. {\frac{\partial N(x,\,t)}{{\partial t}}} \right|_{t = 0} = 0\,.$$

We can get the following coupled non-local system of ODEs after applying the Laplace transform on Eqs. (6)−(9):

12 $$({\text{D}}^{2} - \alpha_{1} )\overline{N} + \varepsilon_{3} \overline{T} = 0,$$

13 $$({\text{D}}^{2} - s)\overline{T} - \alpha_{2} \overline{N} + \alpha_{3} s\,{\text{D}}\overline{u} = 0,$$

14 $$({\text{D}}^{2} - {\mathbb{R}})\overline{u} - {\mathbb{Z}}\,{\text{D}}(\overline{T} + \overline{N}) = 0,$$

15 $$\sigma = \overline{\sigma }_{xx} = \alpha_{4} ({\text{D}}\overline{u} - \overline{T} - \overline{N}),$$

where

$$\text{D} = \frac{\text{d}}{{\text{d}x}}\,,\,\,\,\alpha \,_{1} = A_{1} s\, + A_{2} ,\,\,\,\,\alpha_{4} = \frac{2\mu + \lambda }{\mu },\,\,{\mathbb{R}} = \frac{{s^{2} }}{{1 + s^{2} \xi_{1}^{2} }},\,\,\,{\mathbb{Z}} = \frac{1}{{1 + s^{2} \xi_{1}^{2} }}.$$

The matrix form is utilized to get the solution of the system of Eqs. (12)−(14), by investigating the eigenvalues and eigenvectors to obtain the main physical variations as the following:

16 $$\frac{{{\text{d}}\vec{Z}}}{{{\text{d}}x}} = R\,\vec{Z},$$

$$\vec{Z} = \left[ {\overline{u}\,\,\,\,\overline{N}\,\,\,\,\,\overline{T}\,\,\,\,\,\frac{{{\text{d}}\overline{u}}}{{{\text{d}}x}}\,\,\,\,\,\frac{{{\text{d}}\overline{N}}}{{{\text{d}}x}}\,\,\,\,\frac{{{\text{d}}\overline{T}\,\,}}{{{\text{d}}x}}} \right],$$

and

17 $$R = \left( \begin{array}{llllll}
0 & 0&0&1&0&0\\
0 & 0&0&0&1&0\\
0 & 0&0&0&0&1\\
{a_{41}} & 0&0&0&{a_{45}}&{a_{46}}\\
0 & {a_{52}}&{a_{53}}&0&0&0\\
0 & {a_{62}}&{a_{63}}&{a_{64}}&0&0\\
\end{array}  \right),$$

where $$a_{41} = {\mathbb{R}},\,\,\,a_{45} = a_{46} = {\mathbb{Z}},\,\,\,\,a_{52} = \alpha_{1} ,\,\,\,\,a_{53} = - \;\varepsilon_{3} ,\,\,\,\,a_{62} = \alpha_{2} ,\,\,\,\,a_{63} = s,\,\,\,\,\,\text{and } a_{64} = - \;\alpha_{3} s$$.

## Eigenvalues and eigenvectors approach of a vector–matrix equation

The roots of the characteristic equation, which can be obtained from solving Eq. (16), represent the eigenvalues of the matrix $R$ can be set as $\lambda = \lambda_{1}$, $\lambda = \lambda_{2}$, $\lambda = \lambda_{3}$, $\lambda = \lambda_{4}$, $\lambda = \lambda_{5}$, $\lambda = \lambda_{6}.$ The characteristic equation can take the following form [25]:

18 $$\lambda^{6} - \Re_{1} \lambda^{4} + \Re_{2} \lambda^{2} - \Re_{3} = 0,$$

19 $$\left. \begin{gathered} \Re_{1} = - \;( - \;a_{41} - a_{52} - a_{63} - a_{46} a_{64} ) \hfill \\ \Re_{2} = a_{41} a_{52} + a_{41} a_{63} + a_{52} a_{63} + a_{46} a_{64} a_{52} - a_{53} (a_{62} + a_{45} a_{64} ) \hfill \\ \Re_{3} = - \;( - \;a_{41} a_{53} a_{62} + a_{41} a_{52} a_{63} ) \hfill \\ \end{gathered} \right\}.$$

The corresponding eigenvectors can take the form $\vec{Q} = [q_{1} ,\,\,q_{2} ,\,\,q_{3} ,\,\,q_{4} ,\,\,q_{5} ,\,\,q_{6} ]^{{\,{\text{T}}}} ,$ according to the eigenvalues $\lambda_{\,i} (i = 1\,,\,\,2\,,\,\,3,\,\,4,\,\,5,\,\,6)$ which can be given as:

20 $$\left. \begin{gathered} q_{1} = \lambda a_{46} ( - \lambda^{2} + a_{52} ) - \lambda a_{45} a_{53} \hfill \\ q_{2} = a_{53} (a_{41} - \lambda^{2} ) \hfill \\ q_{3} = - \,\,(\lambda^{2} - a_{52} )(\lambda^{2} - a_{41} ) \hfill \\ q_{4} = \lambda \,Q_{1} \hfill \\ q_{5} = \lambda \,Q_{2} \hfill \\ q_{6} = \lambda \,Q_{3} \hfill \\ \end{gathered} \right\}.$$

In this case, the vector solution of the physical variations of our problem can take the following form:

21 $$\overline{Z}(x,\,s) = \,\sum\limits_{i = 1}^{3} {B_{i} \,} \vec{Q}_{i} \,\text{e}^{{ - \lambda_{i} x}} .$$

The main physical fields, according to the linearity, can be obtained as follows:

22 $$\overline{u} = \,\sum\limits_{i = 1}^{3} {B_{i} \,} q_{{_{1} }}^{i} \text{e}^{{ - \,\lambda_{i} x}} \text{,}$$

23 $$\overline{N} = \,\sum\limits_{i = 1}^{3} {B_{i} \,} q_{{_{2} }}^{i} \text{e}^{{ - \,\lambda_{i} x}} \text{,}$$

24 $$\overline{T} = \,\sum\limits_{i = 1}^{3} {B_{i} \,} q_{{_{3} }}^{i} \text{e}^{{ - \,\lambda_{i} x}} \text{,}$$

25 $$\overline{\sigma } = \overline{\sigma }_{xx} \, = \,\, - \;\alpha_{4} \sum\limits_{i = 1}^{3} {B_{i} \,} (\lambda_{i} \,q_{{_{1} }}^{i} + a_{1} \,q_{{_{2} }}^{i} \, + a_{2} {\kern 1pt} q_{{_{3} }}^{i} )\text{e}^{{ - \,\lambda_{i} x}} .$$

## Boundary conditions

The linear expansion summation can determine the behavior of the physical fields with chosen constants. $B_{i} ,$ which can be determined by using the boundary conditions on the surface of the stable non-local medium at $x = 0$ as following:

26 $$ {\overline{u}}\left( {0,s} \right) = b,\quad b >0\quad {\overline{N}}\left( {0,s} \right) = \frac{\mathchar'26\mkern-10mu\lambda}{{s\,D_{e}}},\quad {\overline{T}}\left( {0,s} \right) = \frac{{\overline{T}}_{0}}{s}. $$

The method Cramer can determine the constants $B_{i}$ by using the following determinants [38]:

27 $$ \left. {\Delta = \left| {\begin{array}{*{20}l} {\begin{array}{*{20}l} {U_{1} } \\ {N_{1} } \\ {T_{1} } \\ \end{array} } &{\begin{array}{*{20}l} {U_{2} } \\ {N_{2} } \\ {T_{2} } \\ \end{array} } & {\begin{array}{*{20}l} {U_{3} } \\ {N_{3} } \\ {T_{3} } \\ \end{array} } \\ \end{array} } \right|,\,\,\,\Delta_{1} = \left| {\begin{array}{*{20}c} n & {U_{2} } & {U_{3} } \\ {\frac{\mathchar'26\mkern-10mu\lambda}{{s\,D_{e} }}} & {N_{2} } & {N_{3} } \\ {\frac{{\overline{T}_{0} }}{s}} & {T_{2} } & {T_{3} } \\ \end{array} } \right|,\,\,\Delta_{2} = \left| {\begin{array}{*{20}l} {U_{1} } & n & {U_{3} } \\ {N_{1} } & {\frac{\mathchar'26\mkern-10mu\lambda}{{s\,D_{e} }}} & {N_{3} } \\ {T_{1} } & {\frac{{\overline{T}_{0} }}{s}} & {T_{3} } \\ \end{array} } \right|,\,\,\Delta_{3} = \left| {\begin{array}{*{20}l} {U_{1} } & {U_{2} } & n \\ {N_{1} } & {N_{2} } &{\frac{\mathchar'26\mkern-10mu\lambda}{{s\,D_{e} }}} \\ {T_{1} } & {T_{2} } &{\frac{{\overline{T}_{0} }}{s}} \\ \end{array} } \right|} \right\}, $$

where

28 $$\left. \begin{gathered} U_{1} = - \lambda_{1} a_{46} ( - \lambda_{1}^{2} + a_{52} ) - \lambda_{1} a_{45} a_{53} \hfill \\ U_{2} = - \lambda_{2} a_{46} ( - \lambda_{2}^{2} + a_{52} ) - \lambda_{2} a_{45} a_{53} \hfill \\ U_{3} = - \lambda_{3} a_{46} ( - \lambda_{3}^{2} + a_{52} ) - \lambda_{3} a_{45} a_{53} \hfill \\ \end{gathered} \right\},\,\,\,\,\,\left. \begin{gathered} N_{1} = a_{53} ( - \lambda_{1}^{2} + a_{41} ) \hfill \\ N_{2} = a_{53} ( - \lambda_{2}^{2} + a_{41} ) \hfill \\ N_{3} = a_{53} ( - \lambda_{3}^{2} + a_{41} ) \hfill \\ \end{gathered} \right\},\,\,\,\,\,\,\left. \begin{gathered} T_{1} = - (\lambda_{1}^{2} - a_{52} )\,(\lambda_{1}^{2} - a_{41} ) \hfill \\ T_{2} = - (\lambda_{2}^{2} - a_{52} )(\lambda_{2}^{2} - a_{41} ) \hfill \\ T_{3} = - (\lambda_{3}^{2} - a_{52} )(\lambda_{3}^{2} - a_{41} ) \hfill \\ \end{gathered} \right\}.$$

In this case, the unknowns $B_{i}$ can be obtained as:

29 $$B_{1} = \frac{{\Delta_{1} }}{\Delta },\quad B_{2} = \frac{{\Delta_{2} }}{\Delta },\quad B_{3} = \frac{{\Delta_{3} }}{\Delta }.$$

## Laplace transform inversion

The inversion of the Laplace transform technique is a precious tool for obtaining the physical main variations in the time domain. The sum of the Riemann approximation has been used to investigate the inversion of Laplace, greatly enhancing the accuracy of the results and letting you work out variations in the primary physical domains. Any function $\prod (x,\,t^{\prime})$ can be transformed from the frequency domain to the time domain, as described in Ref. [9]. References [33, 34] provides more details about Laplace transform inversion.

30 $$\prod (x,\,t^{\prime}) = L^{ - 1} \{ \overline{\prod }(x,s)\} = \frac{1}{{2{\uppi \text{i}}}}\int_{{n - {\text{i}}\infty }}^{{n + {\text{i}}\infty }} {{\text{e}}^{{st^{\prime}\,\,}} } \overline{\prod }(x,s){\text{d}}s,$$

where $n,\,m \in R,\,\,\,s = n + {\text{i}}m,$ and ${\text{i}} = \sqrt { - 1}$, Eq. (30) can be rewritten as:

31 $$\prod (x,t^{\prime}) = \frac{{{\text{e}}^{nt} }}{{2{\uppi }}}\,\int\limits_{ - \,\infty }^{\infty } {{\text{e}}^{{{\text{i}}\beta t}} \,} \overline{\prod }(n + {\text{i}}\beta ){\text{d}}\beta .$$

The expansion of Fourier is used to expand the variables for large integers, which are chosen free, the interval $\left[ {0,2t^{\prime}} \right]$ yields:

32 $$\prod (x,\,t) = \frac{{{\text{e}}^{{nt^{\prime}}} }}{{t^{\prime}}}\left[ {\frac{1}{2}\overline{\prod }(x,\,n) + Re\sum\limits_{k = 1}^{N} {\overline{\prod }\left( {x,\,n + \frac{{{\text{i}}k{\uppi }}}{{t^{\prime}}}} \right)( - 1)^{n} } } \right],$$

where $Re$ is defined as the real part of the function.

## Stability analysis of the main variations

### Initial case

The steady or stable solutions of the optical, thermal, and elastic waves in a non-local medium are $\overline{T}(x),\,\,\overline{N}(x),\,\,\overline{u}(x)$, when the system approaches infinite time, stable solutions are achieved. As time diminishes, the system variables vibrate solely along the *x*-axis, signifying a state of equilibrium. In this case, the system of equations can take the following form [38]:

33 $$\left( {\frac{{\text{d}^{2} }}{{\text{d}\,x^{2} }} - A_{2} } \right)\,N + \;\varepsilon_{3} \,T = 0,$$

34 $$\frac{{\text{d}^{2} T}}{{\text{d}{\kern 1pt} x^{2} }} - \alpha_{2} N = 0,$$

35 $$\frac{{\text{d}^{2} u}}{{\text{d}{\kern 1pt} x^{2} }} - \frac{{\text{d}T}}{{\text{d}{\kern 1pt} x}}\, - \,\frac{{\text{d}N}}{{\text{d}{\kern 1pt} x}} = 0,$$

36 $$\sigma { = }\,\sigma_{xx} = \left[ {\frac{{\text{d}{\kern 1pt} {\kern 1pt} u}}{{\text{d}{\kern 1pt} x}} - (\,T\, + N)} \right].$$

Achieving a steady state, also known as a stable state, is crucial for understanding the propagation of displacement distribution (elastic waves), carrier intensity (plasma waves), and temperature distribution (thermal waves), which can be derived as follows:

37 $$\overline{T}(x) = \frac{{T_{0} \sinh {\kern 1pt} \left( {\sqrt {\,\alpha_{2} } \,(x - h)} \right)}}{{\sinh {\kern 1pt} \left( {\sqrt {\,\alpha_{2} } \,h} \right)}},$$

38 $$\overline{N}(x) = \frac{{\sinh \left( {\sqrt {A_{2} } (h - x} \right)\left( {A_{2} N_{0} - \alpha_{2} N_{0} - \varepsilon_{3} T_{0} } \right)}}{{\left( {A_{2} - \alpha_{2} } \right)\,\sinh \left( {\sqrt {A_{2} } \,\,h} \right)}} - \frac{{\sinh \left( {\sqrt {\alpha_{2} } (h - x)} \right)\,\varepsilon_{3} T_{0} \,}}{{\sinh \left( {\sqrt {\alpha_{2} } \,\,h} \right)\,\left( { - A_{2} + \alpha_{2} } \right)\, }},$$

39 $$\frac{{\text{d}\,\overline{u}(x)}}{{\text{d}\,x}} = \frac{{T_{0} \sinh \left( {\sqrt {\alpha_{2} } (h - x)} \right)\,\,}}{{\sinh \left( {\sqrt {\alpha_{2} } \,\,h} \right)\,\,}} + \frac{{\sinh \left( {\sqrt {A_{2} } (h - x)} \right)\left( {A_{2} N_{0} - \alpha_{2} N_{0} - \varepsilon_{3} T_{0} } \right)}}{{\left( {A_{2} - \alpha_{2} } \right)\,\sinh \left( {\sqrt {A_{2} } \,\,h} \right)}} - \;\frac{{\sinh \left( {\sqrt {\alpha_{2} } (h - x)} \right)\,\varepsilon_{3} T_{0} \,}}{{\sinh \left( {\sqrt {\alpha_{2} } \,\,h} \right)\,\left( { - A_{2} + \alpha_{2} } \right)\,}} - \frac{{p_{0} }}{{\alpha_{4} }},$$

where

40 $$\left. \begin{gathered} \overline{\sigma }_{xx} (0)\, = 0,\,\,\,\,\,\,\text{and}\,\,\,\,\,\,\frac{{\text{d}{\kern 1pt} {\kern 1pt} \overline{u}\,(0)}}{{\text{d}{\kern 1pt} x}} = N_{0} + T_{0} , \hfill \\ \,\overline{T}(0) = T_{0} ,\,\,\;\,\overline{T}(h) = 0,\,\,\,\text{and}\,\,\,\,\overline{N}(0) = N_{0} ,\,\,\,\,\overline{N}(h) = 0 \hfill \\ \end{gathered} \right\}.$$

### Method of homotopy perturbation

By employing the principles of homotopy perturbation, we can effectively analyze the disruptions in physical fields. $\tilde{u}\,(x),\,\,\tilde{T}\,(x),\,\,\tilde{N}\,(x)$ and the fluctuations associated with reaching a stable state $\overline{u}\,(x),$ $\overline{T}\,(x),\,\,\overline{N}\,(x).$ This can be achieved using the following relationships:

41 $$\left. \begin{gathered} u\,(x,\,t)\, = \;\overline{u}\,(x)\, + \tilde{u}\,(x)\,{\text{e}}^{ - \;\omega \,t} \hfill \\ T\,(x,\,t)\, = \;\overline{T}\,(x)\, + \,\tilde{T}\,(x)\,{\text{e}}^{ - \;\omega \,t} \hfill \\ N\,(x,\,t)\, = \;\overline{N}\,(x)\, + \,\tilde{N}\,(x)\,{\text{e}}^{ - \;\omega \,t} \hfill \\ \end{gathered} \right\},$$

such that the perturbations $$\tilde{u}\,(x),\,\,\tilde{N}\,(x)\,\,\text{and}\;\,\tilde{T}\,(x)\,$$ consider very small concerning $$\overline{u}(x),$$$$\overline{N}\,(x)$$ and $$\overline{T} \,(x)$$. $$\omega$$ represents the eigenvalues of the perturbation case.

The exponential perturbation form is a mathematical function that can describe the growth or decay of any function, which means it’s suitable to describe our phenomenon. Achieving stability for the physical perturbation quantities during the optoelectronic non-local medium depends on the exponential term in Eq. (41). The exponential term will tend to zero ${\text{e}}^{ - \;\omega \,t} \to 0\,$ as $\,t \to \infty$ considering this fact, the eigenvalue must be greater than zero $\omega> 0.$

Substituting Eq. (41) into Eqs. (6)–(9) the new system of equations in perturbation form can take the following form:

42 $$\frac{{\text{d}^{2} \tilde{u}(x)}}{{\text{d}{\kern 1pt} x^{2} }} - \omega^{2} \tilde{u}(x) + \xi_{1}^{2} \omega^{2} \frac{{\text{d}^{2} \tilde{u}(x)}}{{\text{d}{\kern 1pt} x^{2} }} - \frac{{\text{d}\tilde{T}(x)}}{{\text{d}{\kern 1pt} x}} - \frac{{\text{d}\tilde{N}}}{{\text{d}{\kern 1pt} x}} = 0,$$

43 $$\frac{{\text{d}^{2} \tilde{N}(x)}}{{\text{d}{\kern 1pt} x^{2} }} + (A_{1} \omega - A_{2} )\tilde{N}(x) + \varepsilon_{3} \tilde{T}(x) = 0,$$

44 $$\frac{{\text{d}^{2} \tilde{T}(x)}}{{\text{d}{\kern 1pt} x^{2} }} + \omega \,\tilde{T}(x) - \alpha_{3} \,\omega \frac{{\text{d}{\kern 1pt} {\kern 1pt} \tilde{u}(x)}}{{\text{d}{\kern 1pt} x}} - \alpha_{2} \tilde{N}\left( x \right)\,\, = 0.$$

With small perturbation boundary conditions:

45 $$\left.{\begin{aligned}& {\tilde{u}}\,(0) = 0,\,\,\,\frac{{\text{d}\tilde{u}\,(0)}}{{\text{d}{\kern 1pt} {\kern 1pt} x}} = 0,\,\,\,\,\tilde{N}\,(0) = 0,\\&\frac{{\text{d}\tilde{N}\,(0)}}{{\text{d}{\kern 1pt} {\kern 1pt} x}} = \varepsilon ,\;\;\tilde{T}\,(0) = T_{0} ,\,\,\,\frac{{\text{d}\tilde{T}\,(0)}}{{\text{d}{\kern 1pt} {\kern 1pt} x}} = 0. \end{aligned}}\right\}$$

HPM is the effective and suitable method to solve the above system and describe the stability of the perturbation functions.

## Discussion of the numerical results

The following physical constants are utilized to obtain stable and unstable numerical solutions for the physical variations between silicon (Si) and germanium (Ge) as examples of non-local semiconductor materials. We can graphically extract the parameters in the SI units as indicated in the prior text to visually illustrate the physical fields and the physical perturbation (Table 1) [9].

**Table 1:** Describe the parameter’s constants in the SI units for silicon (Si) and germanium (Ge)

Name (unit)	Symbol	Si	Ge
Lamé’s parameters (N/m^2^)	$\lambda$ $\mu$	$6.4\times 1{0}^{10}$ $6.5\times 1{0}^{10}$	$0\text{.48}\times 1{0}^{11}$ $0\text{.53}\times 1{0}^{11}$
Density (kg/m^3^)	$\rho$	$2330$	$5300$
Absolute temperature (K)	${T}_{0}$	$800$	$723$
Photogenerated carrier lifetime ($\text{s}$)	$\tau$	$5\times 1{0}^{-5}$	$\text{0.4}\times 1{0}^{-6}$
Parameter of electronic distortion (m^3^)	${d}_{n}$	$-9\times 1{0}^{-31}$	$-6\times 1{0}^{-31}$
Carrier diffusion (m^2^/s)	${D}_{e}$	$2.5\times 1{0}^{-3}$	${10}^{-2}$
Energy bandgap (eV)	${E}_{g}$	$1.11$	$0.72$
Linear thermal expansion factor (K^−1^)	${\alpha }_{t}$	$4.14\times 1{0}^{-6}$	$3.4\times 1{0}^{-3}$
Thermic conductance of the substrate (W/(m·K))	$k$	$150$	$60$
Specific heat at persistent strain (J/(kg·K))	${C}_{e}$	$695$	$310$
Recombination speeds (m/s)	$s$	$2$	$2$

### Non-local parameter effect

**Fig. 1:**
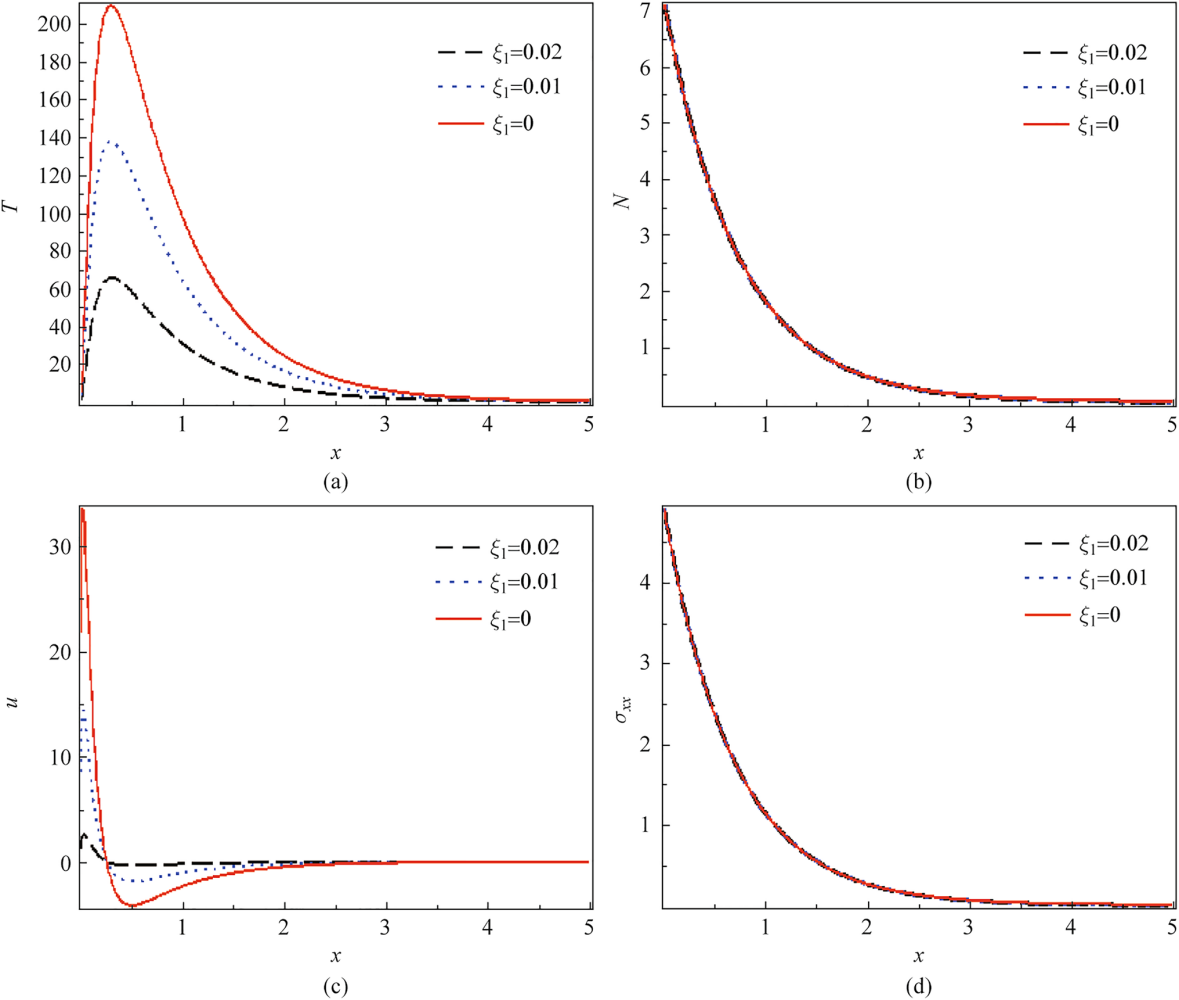
Effect of three values of the non-local parameter on **a*** T*, **b** *N*, **c** *u*, and **d** $\sigma_{xx}$ via *x*

Figure 1a to d represents the variation of the physical quantities (thermal, plasma, mechanical stress, and displacement) opposite the spatial variable according to three cases of the non-local factor namely $\xi_{1} = 0,$ $\xi_{1} = 0.01,\,\,\,\xi_{1} = 0.02.\,\,$ As shown in Fig. 1, the effect of the three different values for the local case and the two non-local cases $\xi_{1} = 0.01,\,\,\,\xi_{1} = 0.02$, show the same behaviors. Figure 1a represents the distribution of the temperature (thermal waves) such that in all three cases, the local and the non-local case. The temperature begins with a positive point satisfying the boundary conditions and increases to arrive at the maximum value, then decreases exponentially to meet the zero line inside the material. The temperature rise followed by exponential decay is what heat conduction does when the semiconductor is subjected to light, which leads to consistency with the photothermal wave propagation theory. As the non-local factor increases, the amplitude of the temperature decreases, which is attributed to the influence of non-local effects on heat transport. This results in a more smoothed temperature profile and diminished amplitude as the non-local factor increases, reflecting a broader thermal diffusion effect. Figure 1b depicts the carrier intensity against the spatial variable *x*-axis. It begins from the high maximum point, achieving the interface of the medium due to the electrons resulting from the photothermal excitation affected by the source light. It decays exponentially far from the surface until it meets the zero line, indicating a minimum value inside the material. It’s noticed that there is no variation of the non-local factor on carrier density at some values. Figure 1c describes the displacement distribution against the dimension variable *x*-axis with the effect of three cases of the non-local factor. As shown in Fig. 1c, the displacement begins its impact from the maximum point satisfying the boundary condition. It sharply decreases to reach the minimum point near the surface, then increases again to get the zero point. We observed that increasing the non-local effect reduces the displacement amplitude, which is attributed to the fact that the non-local effects spread the deformation over a larger region. This result shows that the interactions between the non-local effects tend to smooth out the spatial variations in displacement. Figure 1d measures the influence of three different values, representing the local and the non-local case of the non-local parameter for the stress distribution (mechanical waves) against the spatial variable *x*-axis. It’s observed that the wave of the stress distribution starts from the maximum positive value and decreases gradually till it stabilizes to the minimum point touching the zero line away from the surface. The fact that mechanical stress and carrier density have no effect by increasing the non-local factor indicates that mechanical waves and opto-electronic waves might be less sensitive to non-local impacts than the temperature and displacement distributions in the photothermal process.

### Discussion of the effect of thermo-electric coupling factor

**Fig. 2:**
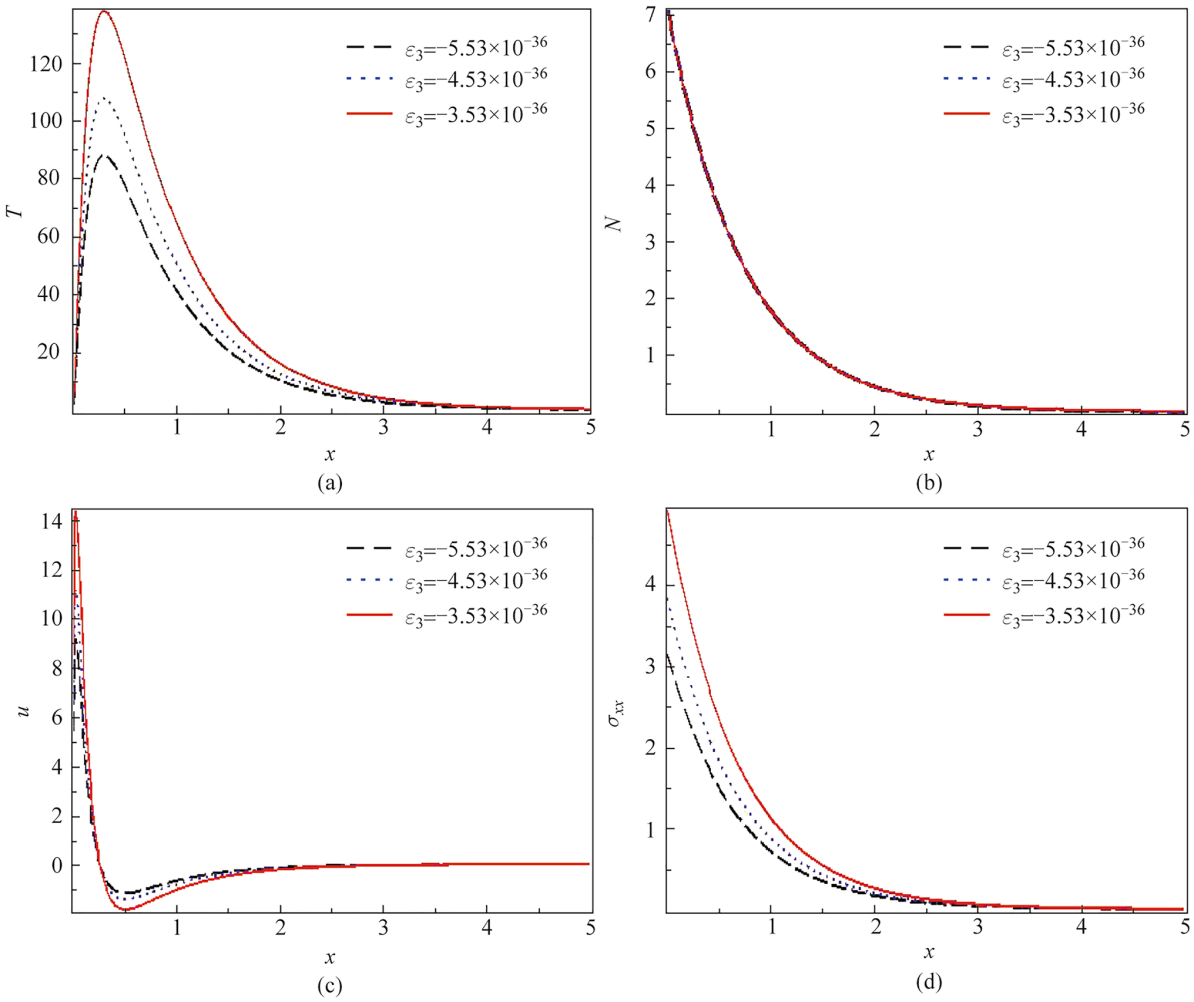
Variation of **a*** T*, **b** *N*, **c** *u*, and **d** $\sigma_{xx}$ via *x* at $\xi_{1} = 0.01$

Figure 2 a to d depicts the variation of different values for the thermo-electric coupling parameter on the physical variations for the thermal waves (temperature distribution), plasma waves (carrier’s distribution), mechanical waves (normal stress), and elastic waves (displacement distribution), versus the horizontal line with the actual physical constants of the silicon (Si) material. Figure 2a recognizes the effect of three physical negative scenarios of the thermo-electric factor on the thermal waves (temperature distribution). From Fig. 2a, it’s noticed that the temperature distribution starts from a positive value, achieving the boundary condition and rising to reach the maximum value, then overlapping to gradually decrease exponentially to meet the zero line, which means the transportation of the temperature inside the material to reach the minimum value as $x \to \infty$. Decreasing the thermo-electric coupling parameter reduces the temperature amplitude, which is logical because the thermo-electric factor impacts how electrical and thermal effects interact. Weaker coupling results in less pronounced temperature variations due to reduced thermal response to electrical excitations. Figure 2b shows that the variation of the thermo-electric coupling parameter does not change at the three different values in the distribution of plasma waves against the horizontal *x*-axis, such that the plasma waves begin from the high maximum positive value satisfying the boundary condition and gradually decrease due to the photothermal excitation process until they reach the minimum value to the zero line. Figure 2c represents the elastic waves (displacement distribution) versus the horizontal line *x*-axis at different values of the thermo-electric coupling parameter. As shown in the figure, the waves of displacement start their distribution from the maximum positive value, achieving the boundary condition. They decrease sharply to arrive at the minimum point near the surface, then increase again to reach the stabilized line (zero line). It’s observed that reducing the thermo-electric coupling factor minimizes the amplitude of the displacement distribution’s behavior. This can be observed by the thermo-electric coupling factor in how thermal and mechanical responses interact. When the coupling is much less, the material’s response to thermal stresses becomes less pronounced, leading to a smaller amplitude of elastic waves. Figure 2d represents the variation of stress distribution (mechanical waves) against the spatial variable *x*-axis with three different negative values of the thermo-electric coupling parameter. The graph shows that the stress wave starts at the maximum positive value due to photothermal excitation. It decreases gradually from the surface to reach the minimum point (zero line). According to Fig. 2d, we can observe that reducing the negative value of the thermos-electric factor leads to dampening the mechanical waves. This fact is logical because the thermos-electric factor is responsible for how the thermal and electrical interactions appear in the material. With reduced coupling, the combined effects of thermal and electrical stresses become less intense, leading to a more subdued stress profile.

### Behavior of two non-local semiconductor materials comparing

**Fig. 3:**
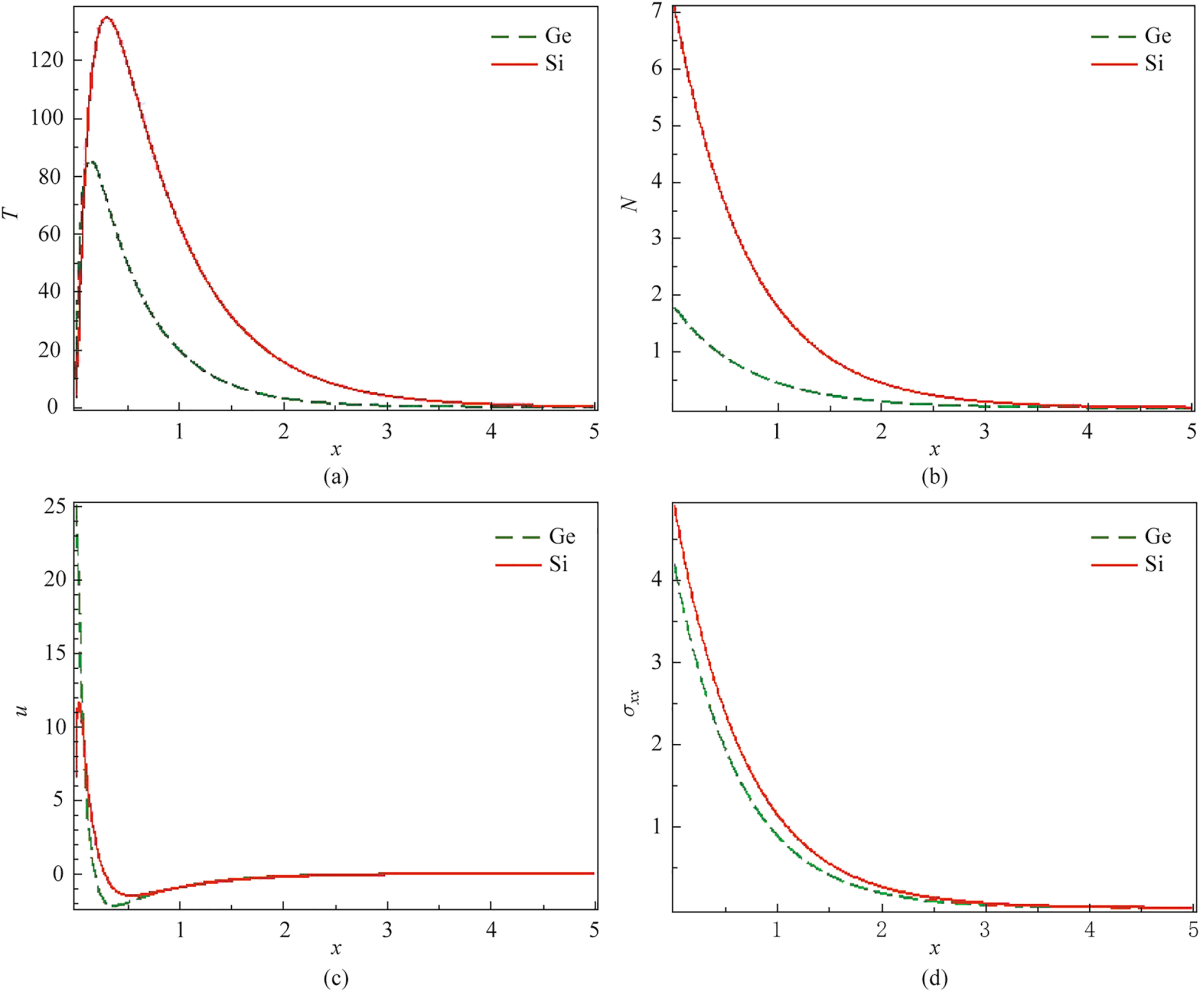
Variation of **a*** T*, **b** *N*, **c** *u*, and **d** $\sigma_{xx}$ for two non-local semi-conductor materials via *x* at $\xi_{1} = 0.01$

Determining how the waves propagate inside the semiconductor material depends on its physical constants, such as thermal, electrical, and elastic properties. Figure 3a–d shows how these constants affect the non-local semiconductor medium’s thermal, plasma, mechanical, and elastic waves. In the context of the PTE theory, the comparison between two semiconductor materials, silicon (Si) and germanium (Ge), is stated graphically. The comparison reveals how variations in these factors influence wave transmission. For example, differences in thermo-electric coupling and non-local effects between Si and Ge can lead to distinct behaviors in wave amplitudes and propagation characteristics. The figures’ visual representation clearly shows how each variable affects wave transmission in both Si and Ge. This detailed analysis helps us understand the fundamental differences between the two semiconductor materials and their performance under different conditions.

### Influence of the eigenvalue approach

**Fig. 4:**
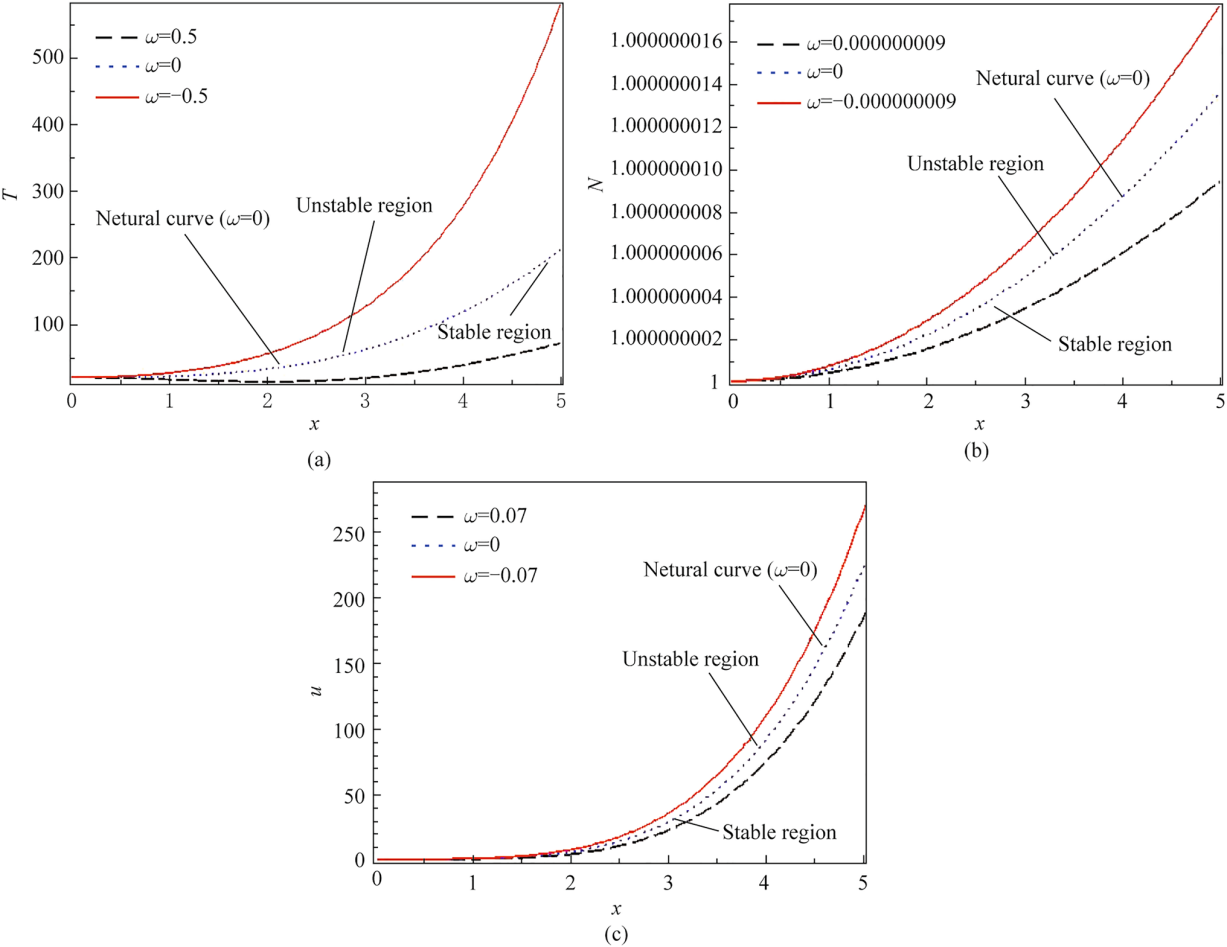
Variation of **a*** T*, **b** *N*, and **c** *u* for different values of the eigenvalue via $x$ at $\xi_{1} = 1$

Figure 4a–c displays the influence of three different values of the eigenvalue on the variation perturbation of physical our quantities versus the spatial variable *x*-axis. All calculations of the physical variables are accomplished in the context of the PTE theory. The perturbation of the physical fields are investigated in a series form using the homotopy stability method. The graph is split into three important regions of study (stable-neutral-unstable). All calculations are accomplished with the physical constants for the silicon (Si) material considering the non-local parameter $$\xi_{1} = 1.$$ As shown in Fig. 4a, the effect of the eigenvalue approach studied on the thermal perturbation against the spatial variable *x*-axis. The illustrated graph shows that the perturbation of the temperature starts from the positive value at the three different cases of the effect of the eigenvalue and increases gradually with increasing the horizontal line *x*-axis according to the influence of perturbation waves. Studying the influence of the eigenvalue at different values on thermal perturbation indicates the maximization (rising) or minimization (damping) of the perturbation of the temperature. When the eigenvalue approach vanishes $$(\omega = 0),$$ it gives the neutral solution (neutral case) of our system between the stable and unstable regions. We have seen that the unstable solution of the system of study appears in the thermal perturbation figure above the neutral curve when the eigenvalue approach $$\omega = - \;0.5$$ and the stable solution of our system of study appear below the neutral case when $$(\omega = 0.5),$$ indicating that the effect of the eigenvalue approach minimize (damping) and stabilize the wave of the thermal perturbation when the eigenvalue getting more positive values. Figure 4b displays the effect of three different values of the eigenvalue approach $$\omega = - \;9 \times 10^{ - \,9} ,\,\,\,\omega = 0,\,\,\,\omega = 9 \times 10^{ - \,9}$$, indicating the (stable-neutral-unstable) solutions of the system of study on the carrier density perturbation against the increasing *x*-axis. It’s observed from the figure that the plasma wave begins from a small positive value and increases with increasing the horizontal line *x*-axis because of the perturbation. It’s noticed from the figure that the transformation of the eigenvalue from the negativity (unstable solution) passing through the zero value (neutral solution) reaching the positivity (stable solution) minimizes the perturbation wave of the plasma wave inside the material. As appeared in Fig. 4c the perturbation of the displacement of the material against the horizontal line *x*-axis is studied with the effect of three different values of the eigenvalue approach $$(\omega = - \;0.07,$$$$\omega = 0,$$$$\omega = 0.07),$$ indicating (stable-neutral-unstable) solutions of our system. It’s observed from the illustrated graph that the perturbation of elastic waves begins from zero value at the three different cases, depicting the boundary condition, and increases with increasing the spatial variable *x*-axis because of the perturbation wave on the physical fields. It’s noticed that the effect of the eigenvalue approach from the negative value (unstable solution) to the positive value (stable solution) minimizes (damping) elastic wave (displacement perturbation).

### Bifurcation solutions of the thermo-electric and thermo-energy factors

**Fig. 5:**
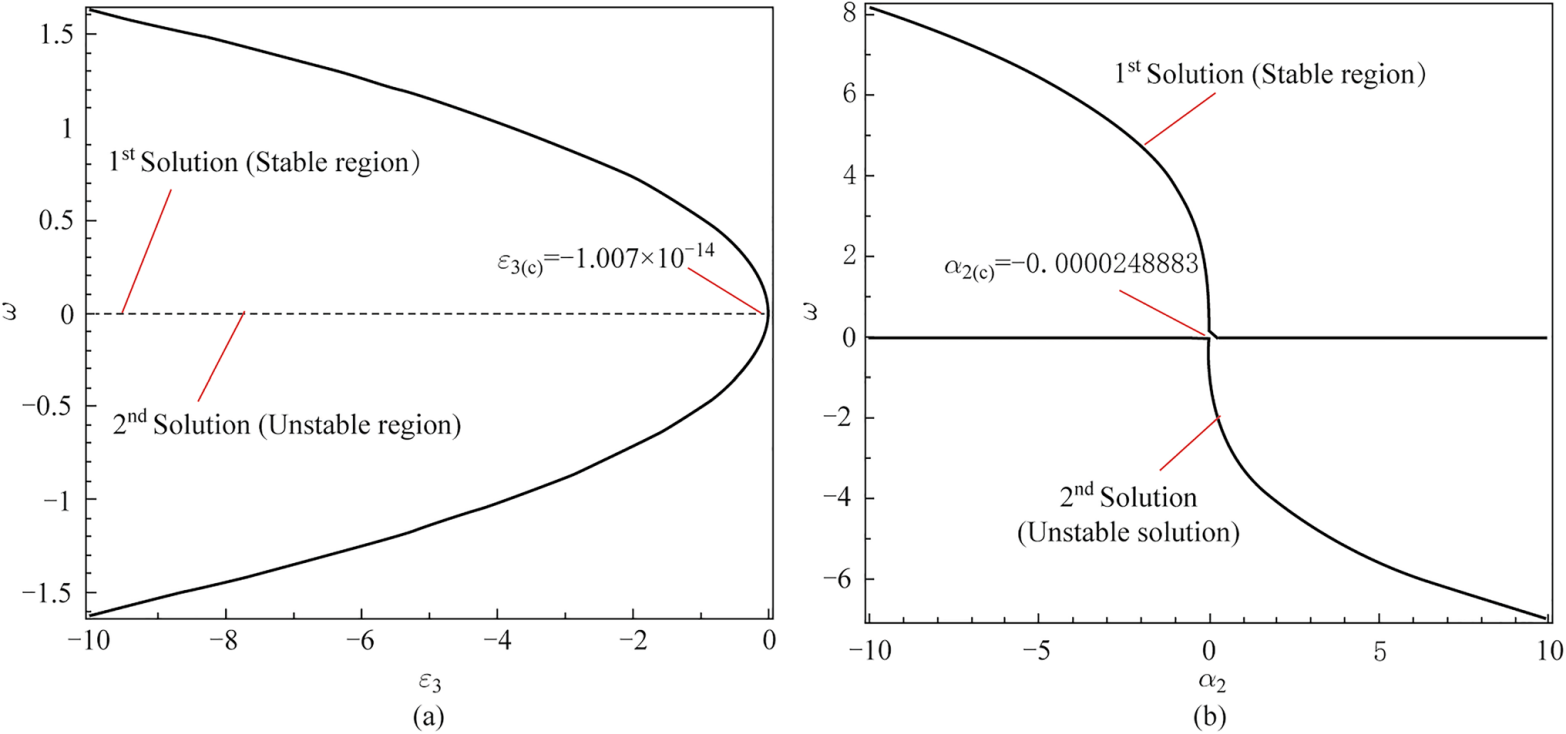
Bifurcation (stable and unstable) solutions between $\omega$ via **a** $\varepsilon_{3}$ and **b** $\alpha_{2}$

Figure 5a, b displays the bifurcation solutions between the eigenvalue and some important factors, such as the thermo-electric factor and thermo-energy coupling parameter, considering the critical points of these factors at neutral case $(\omega = 0).$ As shown in Fig. 5a, the eigenvalue approach is illustrated graphically against the thermo-electric coupling parameter to study the dual solutions for that parameter. The calculation is investigated using the homotopy perturbation method to give the eigenvalue approach as a series function in the thermo-electric coupling parameter. It’s noticed that when the eigenvalue vanishes $(\omega = 0),$ it gives the critical value for the studied (thermo-electric) parameter. The stable and unstable solutions appear in the graph, such that the first solution (stable solution) of the parameter appears when the physical values of the parameter (negative values) meet the positive values of the eigenvalue, meaning the upper solution, conversely, the second solution (unstable solution) is the lower curve in the graph. Figure 5b represents the dual solutions of the eigenvalue approach against the thermo-energy coupling parameter, considering the critical value of the parameter at $\omega = 0.$ The stable region appears when the physical values of the thermo-energy parameter appear in front of the positive values of the eigenvalue as shown in the first solution (stable solution). The second solution (unstable solution) of the parameter appeared below the critical value of the thermo-energy coupling parameter.

### 3D Novel stability study of the stress distribution (mechanical waves)

**Fig. 6:**
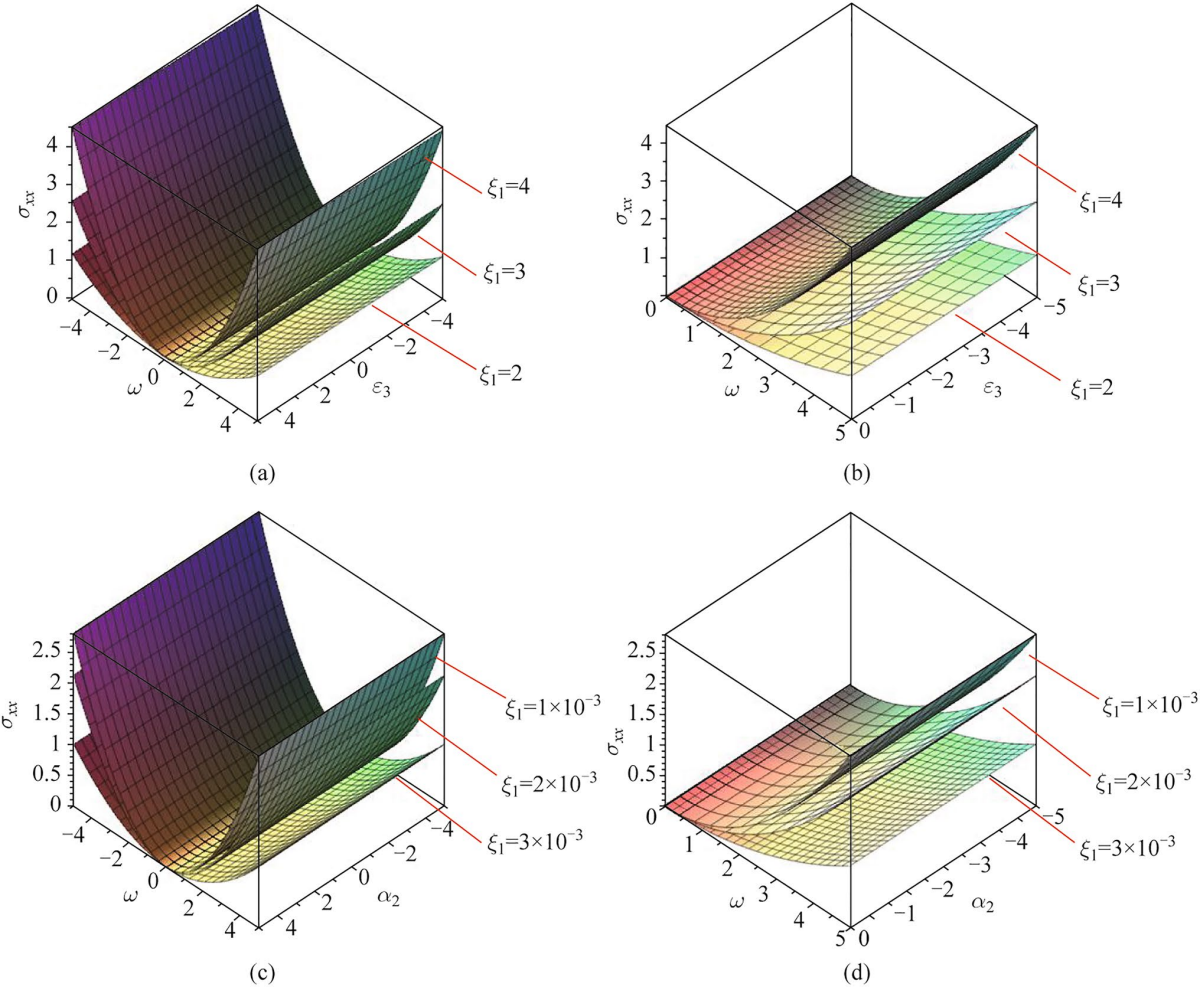
**a** Dual solutions for mechanical stress via $\omega$ and $\varepsilon_{3}$. **b** Stable solution for mechanical stress via $\omega$ and $\varepsilon_{3}$. **c** Dual solutions for mechanical stress via $\omega$ and $\alpha_{2}$. **d** Stable solution for mechanical stress via $\omega$ and $\alpha_{2}$

In this section, the stability perturbation of the stress distribution (mechanical waves) is studied with the influence of three cases for the non-local parameter as shown in Fig. 6a–d. It’s observed that any point in the two dimensions *x*-axis and *y-*axis that represents the bifurcation solutions (unstable and stable) between the thermo-electric factor and the eigenvalue gives the stress function in 3D (*z-*axis) as shown in Fig. 6a according to the definition of the stable solution, the stress perturbation wave (mechanical wave distribution) is stable when the thermo-electric coupling parameter takes its physical values (negative values). The eigenvalue takes the positive values as described in Fig. 6b. It’s observed that the effect of the non-local parameter by decreasing its value led to minimizing (damping) the wave of the stress perturbation in the stable region. Figure 6c displays the stress–strain perturbation in 3D against the dual solution (stable and unstable) between the thermo-energy coupling parameter and the eigenvalue in the plane *x*-axis and *y-*axis with the effect of the non-local parameter at three different values. Choosing any order pairs in the plane (point) in the plane gives the stress perturbation function in the *z-*axis. Figure 6d discusses the novel stable solution of the stress–strain component when the thermo-energy coupling parameter takes its physical values (negative values), and the eigenvalue takes its values (positive values). The illustrated graph shows that increasing the non-local parameter minimizes (damps) the stable region of the stress perturbation.

## Conclusion

This study used non-local semiconductor materials to combine the relationship between photothermal concepts and thermoelastic theory. The Homotopy perturbation method has been employed to obtain the stability analysis for the primary physical variations and the critical parameters, such as thermo-electric and thermo-energy factors. The investigation has shed light on temperature, normal stress, carrier density, and displacement behavior in time-independent and time-dependent scenarios. One can get a stable solution (stable region) for any parameter if the eigenvalue approach’s positive values meet the parameter’s physical values. Furthermore, Investigating the stable case of the primary physical perturbation can be determined when the time-dependent $(t \to \infty ),$ and the eigenvalue takes positive values in exponential form. This investigation concluded that damping the temperature, mechanical stress, carrier density, and displacement waves can result in a stable solution. This new study explores the importance of stabilizing the material to control the waves, leading to more qualified and practical applications of the semiconductor material. We are on the cusp of a new study that will enable us to create more efficient semiconductor materials if they are also studied in the following investigation [11–15]. This study has many applications across various fields in material science, and it contributes to the development, understanding and testing of new semiconductor materials behaviors and enhanced thermal and optical properties. In the field of microsystems, the findings are critical for improving the reliability and performance of MEMS (Micro-Electro-Mechanical Systems) and NEMS (Nano-Electro-Mechanical Systems). PTE stability in semiconductor materials is crucial because it ensures reliable performance and predictable behavior under varying thermal and optical conditions. Stability analysis helps understand how temperature changes and mechanical stresses interact within the material, which is vital for designing semiconductor devices resilient to environmental fluctuations. In the future, scientists could explore stability analysis in two dimensions on a plane using the homotopy perturbation method, focusing on factors like magnetic fields, rotation, and initial stress. This approach could utilize advanced PTE theories, such as the Moore–Gibson–Thompson theory with moisture diffusion, under the influence of the two-temperature theory. This would provide a new perspective on improving applications for semiconductor materials. For instance, semiconductor materials are crucial in developing faster and more efficient electronic devices, solar cells with higher energy conversion rates, and sensors that operate reliably under varying thermal conditions. By understanding their behavior under different physical conditions, researchers could enhance these applications, leading to advancements in technology and materials science.

## Data Availability

The data that support the findings of this study are available from the corresponding author, upon reasonable request.
